# Peripheral neuropathy is associated with insulin resistance independent of metabolic syndrome

**DOI:** 10.1186/s13098-015-0010-y

**Published:** 2015-03-03

**Authors:** Ling Han, Lijin Ji, Jing Chang, Jian Wen, Wenting Zhao, Hongli Shi, Linuo Zhou, Yiming Li, Renming Hu, Ji Hu, Bin Lu

**Affiliations:** Department of Endocrinology and Metabolism, Huashan Hospital, Fudan University, No, 12 Wulumuqi Middle Road, Jing’an District, Shanghai 200040 China; Department of Endocrinology, the Second Affiliated Hospital of Soochow University, Jiangsu, 215004 China; Department of the Third Internal Medicine, Affiliated hospital of Shandong Academy of Medical Sciences, No. 38 Wuyingshan Road, Shandong, 250031 China

**Keywords:** Peripheral neuropathy, Dysglycemia, Insulin resistance, Metabolic syndrome

## Abstract

**Background:**

To determine the association of insulin resistance, metabolic syndrome (MetS) with peripheral neuropathy (PN).

**Methods:**

This cross-sectional study consisted of 2035 subjects in Shanghai who were classified as with MetS and without MetS. The new International Diabetes Federation (IDF) criterion was used to define MetS. HOMA-IR was applied to evaluate insulin resistance. All subjects underwent complete foot examination. PN was assessed according to the neuropathy symptom and neuropathy disability scores. Binary logistic regression was performed to analyze the contributions of insulin resistance, features of MetS to PN.

**Results:**

(1) The percentage of PN was 4.0% in our study. Patients with MetS (47.7%) had a higher percentage of PN (5.5% vs. 2.6%, respectively, P = 0.001). With the components of MetS increased (non-MetS, three, four, five), a linear increase in the proportion of peripheral neuropathy was observed (2.6%, 4.8%, 5.6% and 7.2%; respectively, P for trend = 0.001). (2) In patients with PN, the average age of patients was significantly older than the corresponding non-PN patients. Waist circumference, fasting blood glucose, HbA1c, proportion of treatment for diabetes and hypertension were significantly higher in PN group compared with non-PN group in MetS patients. (3) The frequency of dysglycemia was the highest in PN patients both with and without MetS (96.2% and 82.1%, P = 0.084). (4) After adjusting for gender and smoking history, the PN was associated with MetS [odds ratio (OR) 2.0; 95% confidence interval (CI) 1.2, 3.2; P = 0.006], and age (OR 1.1; 95% CI 1.1, 1.1; P < 0.001). When HOMA-IR was added to this binary logistic regression, the association of PN with MetS disappeared (P = 0.110), but the PN was still associated with HOMA-IR (OR 1.2; 95% CI 1.1, 1.4, P < 0.001).

**Conclusions:**

In metabolic syndrome, insulin resistance might play an important role in the development of peripheral neuropathy.

## Background

Peripheral neuropathy (PN) is a prevalent disorder of the peripheral nervous system, which may be associated with varying combinations of weakness, autonomic changes, and sensory changes. Recently, we reported the slight increase of prevalence of PN in impaired glucose regulation (IGR) subjects, and the higher prevalence of PN in type 2 diabetic subjects and older people [1,2]. As an independent risk factor, plasma glucose level may be an important target for strategies to prevent or improve PN [1].

Metabolic syndrome (MetS) is a cluster of dangerous heart attack risk factors: glucose intolerance, central obesity, hypertension and dyslipidemia. People with MetS are at an increased risk for cardiovascular disease and for increased mortality [3]. Recently, several studies showed that the components of MetS had obvious impact on the pathogenesis of PN [4-8].

Insulin resistance is the core feature of MetS. Data was accumulating to suggest that neurons could also develop insulin resistance, resulting in neuronal injury [4,9]. The components of MetS and insulin resistance both might play an important role in the development of PN. However, few data about the influence of insulin resistance, MetS and its individual components on PN was available in Chinese-based population. Therefore, the aim of this study was to evaluate the association of insulin resistance and MetS with PN in a cross-sectional study.

### Research design and methods

#### Study population

The sample population was recruited from the Shanghai diabetic neuropathy epidemiology and molecular genetics study (SH-DREAMS) from July 2011 to May 2012. SH-DREAMS was a population-based cross-sectional study. All non-pregnant community members aged > 25 years without type 1 diabetes (diagnosed in the past medical history) or renal failure (estimated glomerular filtration rate < 15 ml/min/1.73 m^2^ or current treatment of kidney dialysis) were invited to participate in our study, and a total of 2149 voluntary individuals were enrolled from 2 communities: Gongkang and Sitang. A total of 114 subjects removed from the study for the following reasons: some difficulties in completing the required tests and the incomplete laboratory measurements. Finally, a total of 2035 subjects, including 728 men and 1307 women, were analyzed. Written informed consents were obtained from all participants. All protocols were approved by Huashan Hospital ethics committee.

#### Anthropometric measurements

All participants were asked to complete a questionnaire to collect their demographic information as well as medical history of diabetes and related diseases. Physical examination included measurements of height, weight, waist circumference, and blood pressure. Blood pressure was measured twice using a standard mercury sphygmomanometer and then averaged. Body mass index (BMI) was calculated as weight divided by height squared (kg/m^2^). Other information such as living habits and lifestyle was also collected.

#### Laboratory measurements

After an overnight fast, venous blood samples were collected. Each participant received a 75-g OGTT (oral glucose tolerance test), except for those with a validated history of type 2 diabetes mellitus with 100 g steamed bread meal test. 100 g steamed bread meal test, though not a standard test in diabetes research or care, can avoid severe glucose fluctuation in those with a validated history of type 2 diabetes mellitus. Fasting blood samples were analyzed for fasting blood glucose, glycated hemoglobin (HbA1c), fasting insulin and lipid profiles including total cholesterol, triglycerides, high density lipoprotein-cholesterol (HDL-C), and low density lipoprotein-cholesterol (LDL-C). Plasma glucose levels were measured using the glucose oxidase method. Levels of HbA1c were measured by high-pressure liquid chromatography using an analyzer (HLC-723G7, Tosoh Corporation, Japan). Lipid profiles were measured on a Hitachi 7600 analyzer using an enzymatic assay. Homeostasis model assessment of Insulin resistance (HOMA-IR) values were estimated as follows: [fasting blood glucose (mmol/L)*fasting insulin (mU/L)]/22.5. HOMA-IR was applied to evaluate insulin resistance.

#### Definitions

MetS was defined by the new International Diabetes Federation (IDF) definition [10,11] if they have three or more of the following: **(1)** abdominal obesity (waist circumference ≥ 90 cm for men and ≥ 80 cm for women); **(2)** triglycerides ≥ 150 mg/l (≥1.7 mmol/l) or current use of medication for dyslipidaemia; **(3)** HDL-C < 1.03 mmol/l (<40 mg/dl) for men or < 1.29 mmol/l (<50 mg/dl) for women or current use of medication for dyslipidaemia; **(4)** blood pressure ≥ 130/85 mm Hg or current use of medication for hypertension; **(5)** fasting plasma glucose ≥ 5.6 mmol/l (100 mg/dl) or current use of medication for diabetes mellitus. Type 2 diabetes and IGR were defined by the ADA definition [1].

#### PN screening and assessment

Neuropathy Deficit Score (NDS): Neuropathic deficits in the feet were determined using the NDS, derived from the examination of vibration (using a 128-Hz tuning fork), pin-prick sensation (using Neurotip), temperature sensation (using warm and cool rods), and Achilles tendon reflex (using a tendon hammer). The three perceptions (vibration sense, pain and temperature sensation) were scored 0 if present and normal, and 1 if absent, reduced, or uncertain. On either side, the ankle reflex was scored 0 if present and normal, and 2 if absent [12]. The maximum score was 10. The severity of neuropathy disability was graded as follows: mild (scores: 3–5), moderate (scores: 6–8), and severe (scores: 9–10).

Neuropathy Symptom Score (NSS): all patients were asked whether they experienced pain or discomfort in their legs. A description of burning, numbness, or tingling was assigned a score of 2, and fatigue, cramping, or aching was assigned a score of 1. If the patient described the symptoms as occurring in their feet, calves, and elsewhere, scores of 2, 1, and 0 were assigned, respectively. Nocturnal exacerbation of symptoms was scored as 2; appearance of symptoms during the day as well as night was scored as 1, and the symptoms arise during the daytime alone was scored as 0. If the symptoms had ever woken the patient from sleep, a score of 1 was assigned. The patients were asked if any maneuver could reduce their symptoms; walking was assigned a score of 2, standing 1, and sitting or lying down 0. Thus, the maximum symptom score was 9, and the severity of symptoms was graded as follows: mild (scores: 3–4), moderate (scores: 5–6), and severe (scores: 7–9) [13].

The diagnosis of PN depended on both subjective symptoms and signs of neuropathy. We defined PN as at least moderate signs with or without symptoms (NDS ≥ 6), or mild signs with moderate symptoms (NDS ≥ 3 and NSS ≥ 5) [1,12,14].

#### Statistical analysis

All data was expressed as mean ± SD or number of cases and percent of individuals affected. A statistical package for SPSS software (version 19.0) was used for statistical analysis. Comparisons between groups were performed using chi-square test or independent t test or Mann–Whitney U test when appropriate. Binary logistic regression analysis was performed to evaluate the risk factors for PN. Spearman correlation analysis was calculated to assess the relationship among insulin resistance, MetS and PN. The p value < 0.05 was considered to be significant.

## Results

A total of 2035 subjects, including 728 men and 1307 women, had an average age of 61.5 ± 10.1 years. Among these 2035 subjects, 970 and 1065 subjects were diagnosed with MetS and without MetS. 458, 1043 and 534 subjects were diagnosed with NGT (normal glucose tolerance), IGR and T2DM (type 2 diabetes mellitus) respectively according to the ADA definition after 75-g OGTT tests.

Table 1 presented the clinical characteristics of study population. Among the five groups (NGT, IGR, T2DM, non-MetS and MetS) of subjects, there were no obvious differences between non-PN and PN subjects in the following variables: smoking history, BMI, diastolic blood pressure, HDL-C, and the proportion of patients taking treatment for dyslipidemia. In patients with PN, the average age of patients was significantly older than the corresponding non-PN patients. Duration of diabetes and the proportion of patients taking treatment for dysglycemia were higher in type 2 diabetic patients with PN than non-PN. Moreover, waist circumference, fasting blood glucose, HbA1c, proportion of treatment for diabetes and hypertension were significantly higher in PN group compared with non-PN group in MetS patients. Additionally, in non-MetS group, patients with PN had a higher level of systolic blood pressure than patients without PN.

**Table 1:** **Clinical characteristics of subjects among NGT, IGR, T2DM, non-MetS and MetS groups**

	**NGT(n = 458)**		**IGR(n = 1043)**		**T2DM(n = 534)**		**no-MetS(n = 1065)**		**MetS(n = 970)**	
	**non-PN(n = 451)**	**PN(n = 7)**	**non-PN(n = 1014)**	**PN(n = 29)**	**non-PN(n = 489)**	**PN(n = 45)**	**non-PN(n = 1037)**	**PN(n = 28)**	**non-PN(n = 917)**	**PN(n = 53)**
Male/All	141/451	0/7	348/1014	9/29	217/489	13/45*	391/1037	9/28	315/917	13/53
Age(year)	60.7 ± 11.0	76.7 ± 9.7*	60.7 ± 9.3	71.4 ± 9.7*	63.5 ± 9.7	69.6 ± 9.5*	60.5 ± 10.2	76.6 ± 10.6*	61.7 ± 9.5	70.5 ± 9.3*
Smoking history	68/451	0/7	134/1014	2/29	78/489	4/45	159/1037	1/28	121/917	5/53
Duration of T2DM(years)	—	—	—	—	4.2 ± 6.3	11.2 ± 9.6*	—	—	—	—
BMI (kg/m2)	23.5 ± 3.1	25.4 ± 4.3	24.3 ± 3.4	24.3 ± 3.9	25.5 ± 3.8	25.8 ± 4.3	23.1 ± 3.0	24.1 ± 4.1	25.9 ± 3.4	25.8 ± 4.1
WC(cm)	82.4 ± 9.1	90.3 ± 11.0	85.4 ± 9.6	88.0 ± 12.2	90.2 ± 10.2	92.5 ± 10.5	81.6 ± 8.9	85.9 ± 12.8	90.8 ± 9.0	93.2 ± 9.5*
SBP (mmHg)	124.1 ± 18.4	136.1 ± 19.5	126.1 ± 18.6	132.4 ± 23.6	135.2 ± 20.9	139.4 ± 23.0	121.5 ± 17.8	130.1 ± 21.6*	135.2 ± 19.1	140.0 ± 23.0
DBP (mmHg)	78.7 ± 10.5	80.6 ± 10.0	79.8 ± 10.2	77.4 ± 12.0	81.7 ± 10.7	79.7 ± 11.0	77.1 ± 9.6	74.6 ± 10.1	83.3 ± 10.4	81.3 ± 11.1
FBG (mmol/L)	4.5 ± 0.6	4.3 ± 0.3	5.4 ± 0.8	5.3 ± 0.8	7.3 ± 2.7	8.1 ± 3.8	5.2 ± 1.5	5.3 ± 1.4	6.2 ± 2.0	7.6 ± 3.6*
HbA1c (%)	5.4 ± 0.2	5.5 ± 0.1	5.8 ± 0.3	5.7 ± 0.4	7.1 ± 1.5	7.5 ± 1.6	5.8 ± 0.8	6.0 ± 0.9	6.3 ± 1.2	7.0 ± 1.7*
TG (mmol/L)	1.5 ± 0.9	1.4 ± 0.7	1.7 ± 1.0	1.4 ± 0.7	2.1 ± 1.6	1.9 ± 1.6	1.3 ± 0.6	1.0 ± 0.4*	2.2 ± 1.4	2.0 ± 1.5
TC (mmol/L)	5.2 ± 1.0	5.4 ± 0.6	5.4 ± 1.0	5.1 ± 0.9*	5.5 ± 1.2	5.5 ± 1.3	5.3 ± 1.0	5.3 ± 1.4	5.5 ± 1.1	5.3 ± 1.0
HDL-C (mmol/L)	1.4 ± 0.3	1.3 ± 0.2	1.4 ± 0.3	1.3 ± 0.4	1.3 ± 0.3	1.2 ± 0.3	1.5 ± 0.3	1.5 ± 0.3	1.2 ± 0.3	1.2 ± 0.3
LDL-C(mmol/L)	3.1 ± 0.7	3.5 ± 0.4	3.1 ± 0.7	2.9 ± 0.6	3.2 ± 0.8	3.3 ± 0.9	3.1 ± 0.7	3.2 ± 1.0	3.2 ± 0.8	3.2 ± 0.7
Treatment of diabetes	—	—	0.2%	3.4%	36.0%	71.1%*	4.0%	17.8%*	14.9%	52.8%*
Treatment of dyslipidemia	2.9%	14.3%	1.1%	0.0%	2.2%	6.7%	0.9%	3.6%	2.8%	5.7%
Treatment of hypertension	22.7%	28.6%	22.8%	44.0%*	43.6%	45.5%	16.0%	35.3%*	41.6%	49.0%

Categorical variables were expressed as numbers or percentages.

Continuous variables were expressed as mean ± SD.

NGT: normal glucose tolerance; IGR: impaired glucose regulation; T2DM: type 2 diabetes mellitus; MetS: metabolic syndrome; PN: peripheral neuropathy; BMI: body mass index; WC: waist circumference; SBP: systolic blood pressure; DBP: diastolic blood pressure; HbA1c: glycated hemoglobin A1c; FBG: fasting blood glucose; HDL-C: high density lipoprotein-cholesterol; LDL-C: low density lipoprotein-cholesterol.

*significantly different from non-PN and corresponding PN groups (P value < 0.05).

The percentage of PN was 4.0% in our study. Patients with MetS (47.7%) had a higher prevalence of PN (5.5% vs. 2.6%, respectively, P = 0.001). PN was correlated with MetS (r = 0.1, P = 0.001) and HOMA-IR (r = 0.1, P = 0.002) by Spearman correlation analysis. A linear increase was observed in the percentage of PN (2.6%, 4.8%, 5.6%, and 7.2%, respectively, P for trend = 0.001) with the increase in the number of MetS components (non-MetS, three, four, five). However, the percentage of PN was not significantly different between men and women (3.0% vs. 4.5%,P = 0.099).

**Figure 1:**
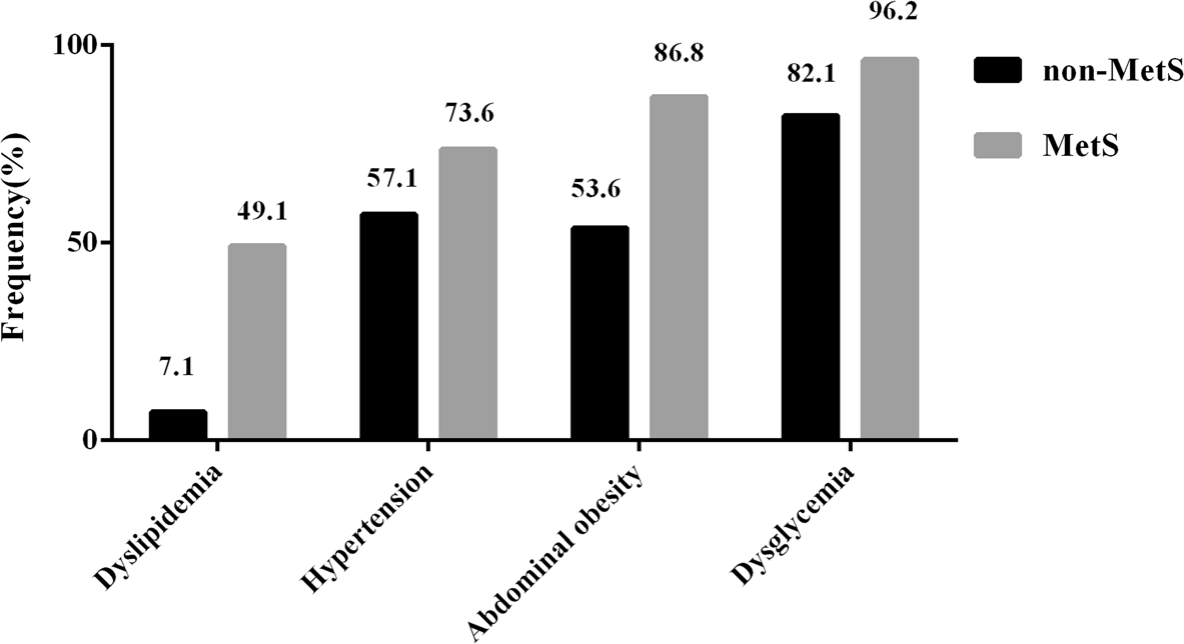
**The frequencies of MetS components in PN patients.** MetS: metabolic syndrome; PN: peripheral neuropathy.

Figure 1 showed the frequencies of MetS components in PN patients. As shown, the frequency of dysglycemia was the highest in PN patients both with and without MetS (96.2% and 82.1%, P = 0.084). The higher frequencies of dyslipidemia (P < 0.001) and abdominal obesity (P = 0.001) in MetS group were also observed than non-MetS group in PN patients.

Binary logistic regression was performed to analyze the contributions of insulin resistance and features of the MetS to PN. HOMA-IR was applied to evaluate insulin resistance. After adjusting for gender and smoking history, the PN was associated with MetS [odds ratio (OR) 2.0; 95% confidence interval (CI) 1.2, 3.2; P = 0.006], and age (OR 1.1; 95% CI 1.1, 1.1; P < 0.001). However, when HOMA-IR was added to this binary logistic regression, the association of PN with MetS disappeared (P = 0.110), but the PN was associated with HOMA-IR (OR 1.2; 95% CI 1.1, 1.4, P < 0.001) (Table 2).

**Table 2:** **Binary logistic regression of peripheral neuropathy** ^**a**^

	**Model 1** ^**b**^				**Model 2** ^**c**^			
	**OR**	**95% CI**		**P value**	**OR**	**95% CI**		**P value**
		**Lower**	**Upper**			**Lower**	**Upper**	
Age	1.1	1.1	1.1	0.000*	1.1	1.1	1.1	0.000*
Gender	1.6	0.9	2.7	0.125	1.6	0.9	2.8	0.122
Smoking history	0.7	0.3	1.9	0.522	0.8	0.3	2.0	0.588
MetS-IDF ^d^	2.0	1.2	3.2	0.006*	1.5	0.9	2.5	0.110
HOMA-IR	**/**	**/**	**/**	**/**	1.2	1.1	1.4	0.000*

^a^Table 2 showed the binary logistic regression results of peripheral neuropathy. ^b^Age, gender, smoking history and MetS-IDF were taken into the logistic regression equation in model 1.^c^ And in model 2, HOMA-IR was added to the equation based on model 1. ^d^MetS-IDF, metabolic syndrome was defined using the new International Diabetes Federation. ^e^*significantly different in the model (p value < 0.05).

## Discussion

Our present study showed the percentage of PN was 4.0% in a community-based Chinese population. The Italian General Practitioner Study Group interviewed 4191 elderly subjects (66.5% were ≥65 years) in northern Italy and found possible neuropathy in 8.0% [15]. In comparison, only 34.4% of this Chinese population was ≥ 65 years. The National Health and Nutrition Examination Survey (NHANES) study revealed a 14.8% prevalence of PN among 2873 individuals aged ≥ 40 years and also observed that MetS affected 34.0% of adults in the US [16,17]. The marked variation in the prevalence of PN might be due to several reasons. Firstly, it was possibly attributed to the wide variation in definitions of PN. In NHANES study, PN was defined as more than one insensate area according to monofilament testing. While in our study, the diagnosis of PN was based on NDS and NSS. Monofilament testing is a simple and convenient method to diagnose PN, but the sensitivity and specificity in diagnosis of PN remain controversial. Secondly, individuals recruited by previous studies had higher frequency of smoking history than subjects observed in our study (47.0% vs. 14.1%), which contributed to a higher prevalence of PN. Thirdly, race difference might cause a variation in prevalence of PN.

Our results showed that the average age in PN group was significantly older than the corresponding non-PN group, and PN was independently associated with age. These results supported evidences that age was an important risk factor for the development of PN. However, the PN definition was based on NDS and NSS in our study, and NDS might be affected by the age-related reduction of lamellar corpuscles at sensory nerve endings and the decrease of cutaneous innervation [18].

The current study presented approximately 47.7% of adults met the criteria for MetS. Compared to the non-MetS group, patients with the MetS had a higher prevalence of PN (5.5% vs. 2.6%). Consistent with Costa’s study [19], a linear increase in the proportion of PN was observed with the increase in the number of MetS components. The results of binary logistic regression in model 1 showed PN was significantly associated with MetS. In MetS patients of our study, fasting blood glucose and HbA1c were significantly higher in PN group compared with non-PN group. Furthermore, among the PN patients, the frequency of dysglycemia was the highest in both non-MetS and MetS groups. Similar to previous studies, dysglycemia showed the strongest evidence supporting a pathogenic link with PN [20-22]. These results revealed glucose dysregulation, contributing to the diagnosis of MetS, was the leading feature associated with the progression of PN.

Additionally,we also found that, even in non-MS subjects, the higher systolic blood pressure level in PN group was observed. Thus, blood pressure might be also a risk factor for PN. Zarrelli reported hypertension was associated with chronic symmetric polyneuropathy after adjustment for a few common causes of polyneuropathy in Italian subjects aged ≥ 55 years [23].

Moreover, PN was proved to be independently associated with HOMA-IR after adjusting for MetS in binary logistic regression in model 2. As mentioned above, dysglycemia, as a central feature of MetS, may play a key role in the development of PN. Insulin is the major anabolic hormone regulating the homeostasis of glucose. Insulin resistance is defined as a state of decreased responsiveness of target tissues to normal circulating levels of insulin and is the central feature of type 2 diabetes and MetS [24]. In insulin-dependent tissues, such as adipose tissue and muscle, the phosphatidylinositol 3-kinase (PI3K)/Akt signaling was crucial for the metabolic effects of insulin, and this pathway was generally affected in individuals with the MetS and diabetes [4,5]. Several researches suggested that insulin was one of the growth factors for neurons, and its receptor was widely expressed in nervous systems [25,26]. Chronic insulin stimulation was shown to induce insulin resistance in mouse DRGs, as evidenced by decreased activation of Akt and its downstream effectors, and could attenuate the neurotrophic effects of insulin, resulting in mitochondrial dysfunction, subsequent development of PN [27]. These findings indicated that in the MetS insulin resistance might play an important role in the development of PN.

There were several limitations in our study. Firstly, the major limitation of the present study was the criteria used to define neuropathy or in particular nerve damage. The NDS and NSS score is the lack of a rigorous assessment of small fibre damage which is thought to be the earliest nerve fibre deficit and the one likely to occur in IGR. However, an important methodological strength of this study was the use of the OGTT. This enabled us to study the entire spectrum of glucose disorders by identifying subjects with undiagnosed diabetes and pre-diabetes. Secondly, because of serious painful symptoms, patients in PN group had higher percentages of treatment for diabetes and hypertension than subjects without PN. Due to this condition, these data could not reflect the original healthy status of patients. Thirdly, we could not make inferences regarding cause and effect because of the cross-sectional design.

## Conclusions

Insulin resistance might play an important role in the development of peripheral neuropathy in the metabolic syndrome.

## Abbreviations

PN: peripheral neuropathy
IR: Insulin resistance
MetS: Metabolic syndrome
HOMA-IR: Homeostasis model assessment of insulin resistance
FBG: Fasting blood glucose
NGT: Normal glucose tolerance
IGR: Impaired glucose regulation
T2DM: Type 2 diabetes mellitus
OGTT: Oral glucose tolerance test
HbA1c: Glycated hemoglobin A1c
HDL-C: High density lipoprotein-cholesterol
LDL-C: Low density lipoprotein-cholesterol
BMI: Body mass index
WC: Waist circumference
SBP: Systolic blood pressure
DBP: Diastolic blood pressure
IDF: International Diabetes Federation
